# Infectious bronchitis-virus-like QX strain transmission, pathogenesis, replication, and host miRNA biogenesis pathway hijacking mechanism

**DOI:** 10.3389/fcimb.2025.1645086

**Published:** 2025-09-01

**Authors:** Asad Khan, Iftikhar Ali Khan, Chao Huang, Qihui Luo, Abdul Qadeer, Lanlan Jia, Mourad A. M. Aboul-Soud, Noorah A. Alkubaisi, Zhengli Chen

**Affiliations:** ^1^ Laboratory of Experimental Animal Disease Model, College of Veterinary Medicine, Sichuan Agricultural University, Chengdu, China; ^2^ Key Laboratory of Animal Disease and Human Health of Sichuan Province, College of Veterinary Medicine, Sichuan Agricultural University, Chengdu, China; ^3^ College of Veterinary Medicine, Nanjing Agricultural University, Nanjing, Jiangsu, China; ^4^ University of Artois, University of Lille, University of Littoral Côte d’Opale, University of Picardie Jules Verne, University of Liège, INRAE, Junia, UMR-T 1158, BioEcoAgro, Lens, France; ^5^ Department of Cell Biology, School of Life Science, Central South University, Changsha, China; ^6^ Center of Excellence in Biotechnology Research, College of Applied Medical Sciences, King Saud University, Riyadh, Saudi Arabia; ^7^ Department of Botany and Microbiology, College of Science, King Saud University, Riyadh, Saudi Arabia

**Keywords:** IBV-QX, intranasal route transmission, ACE2 receptor, miRNA biogenesis pathway, RNA-interference

## Abstract

The infectious bronchitis virus (IBV) is an acute, highly contagious, single-stranded RNA (ssRNA) gammacoronavirus mainly transmitted to chickens through the intranasal route. Positive-sense ssRNA viruses primarily act on mRNA and enhance the replication of viral copies. We identified the nasal entry site of the IBV-QX strain and provided insights into the minimal viral replication in systemic organs. Here is an overview of its entry mechanisms and tropism in systemic organ tissues. It enters the host cells via spike proteins, which bind to highly expressed receptors in respiratory, renal, and gastric epithelial cells. Viral RNA primarily replicates in the host cell environment, where it is directly translated into viral proteins. The precise replication of the IBV-QX strain in gastric epithelial cells was previously unknown. Different IBV strains have varying tropism. For the first time, we revealed the key players involved in the microRNA (miRNA) biogenesis pathway by transfecting gastric cells with the IBV-QX strain. Our findings suggest that the QX strain may bind to angiotensin-converting enzyme-2 (ACE2) receptors by circulating throughout the lymphatic system at the very least and influence the translation of argonaute2 (AGO2), Dicer, exportin5 (XPO5), and Drosha proteins. Taken together, QX viral proteins disrupt host miRNA biogenesis, leading to dysregulated immune and cellular responses that enhance viral replication and systemic spread, thereby enabling cross-organ tropism and multi-system pathogenesis.

## 1 Introduction

Infectious bronchitis (IB) is a highly contagious viral respiratory disease affecting chickens and caused by infectious bronchitis virus (IBV) (Cook et al., 2012). This virus is classified as a Gamma-coronavirus, which belongs to the *Coronaviridae* family and possesses ssRNA genome characteristics (Cavanagh, 2005). IBV primarily targets the respiratory tract epithelial cells, yet it can also infect other organs depending on the strain. New variants of IBV are continually being reported, leading to the emergence of different genotypes, serotypes, and pathotypes (Valastro et al., 2016). The QX genotype (GI-19) of IBV is indeed one of the most concerning strains globally due to its widespread prevalence, high pathogenicity, and ability to evade conventional vaccine protection. The first QX strain was reported in China and is now circulating in various countries, especially in Europe, as the second most prevalent genotype (Yudong et al., 1998; Worthington et al., 2008; de Wit et al., 2018).

The coronaviruses possess a (~26–32 kb) positive-sense RNA genome, which is one of the largest among RNA viruses that cause various significant diseases affecting both humans and animals (Holmes, 1999). Defending against coronavirus infections is challenging for the host due to several biological and immunological factors (Keep et al., 2022). The QX-RBD recognizes different sialylated structures and suggests that they may target different cell or tissue types (Cook et al., 2012; Bartel, 2018). Reciprocal mutations in QX-RBD are detrimental (Bouwman et al., 2020). Variations in viral protein attachment alter glycan-binding specificity, thereby redirecting the virus to new tissues or hosts. The QX-RBD mutations may optimize binding to ACE2, and reverting them weakens this interaction. Research on identifying specific regulators to develop and establish human gastric cell lines is essential for maintaining gastric functions and understanding their underlying mechanisms (Basso et al., 2010; De Reuse and Bereswill, 2007). Viruses can influence the host cell cycle to enhance their replication (Benedict et al., 2002; Hay and Kannourakis, 2002).

RNA interference (RNAi) is a highly conserved regulatory gene mechanism in eukaryotes mediated by small non-coding RNAs (Baulcombe, 2005). The main functions of RNA silencing serve as a crucial innate defense mechanism against viral infections (Ding, 2010). A variety of genetic studies have identified key protein components necessary for RNA silencing pathways. In the context of antiviral silencing, the Dicer-like (DCL) protein plays a critical role. They act as the primary sensors for detecting viral RNA and processing it into primary small interfering RNAs (siRNAs) that drive the cleavage of corresponding viral RNAs by integrating them into an (AGO) effector complex. DCL proteins play a crucial role in processing viral single-stranded siRNAs into double-stranded RNA (dsRNA). However, to amplify the antiviral silencing response, the host employs RNA-dependent RNA polymerases (RDRs) to enhance siRNA production (Wang et al., 2010). Pattern recognition receptors (PPRs) usually have two important functional domains. One domain actively engages with microbial signatures common across major microbial classes, while the second protein–protein interaction domain initiates crucial downstream signaling events. This process ultimately stimulates the transcription of immunity effector genes, which possess powerful, broad-spectrum antimicrobial activities essential for effective defense (Ishii et al., 2008). This concept is critical to understand viral evolution, zoonotic spillover, and therapeutic designs. The IBV-QX strain might use ACE2 as its primary entry receptor and disrupt miRNA biogenesis in gastric cells. This interference inhibits or dysregulates key translational and functional processes, ultimately driving viral pathogenesis and compromising host cell activity.

## 2 Materials and methods

### 2.1 Experimental model design and sample collection

Randomly assigned to either the control or the infected group were 3-week-old specific pathogen-free (SPF) chicks. Chicks from the infected groups were carefully inoculated intranasally with the IBV-M41 and QX strains. In contrast, the control group was administered PBS to ensure a valid comparison. Both infected and control chicks were sacrificed at 6, 12, 24, and 48 h post-infection (hpi) based on the different experimental requirements. Samples of nasal, trachea, lungs, kidneys, proventriculus, spleen, duodenum, colon, and peripheral blood monocytes (PBMCs) were collected from each group. All tissue samples were obtained for histopathology and mRNA and protein examination. However, PBMC samples were used for flow cytometry analysis.

### 2.2 Reagents and antibodies

Histopaque-1077, which has a density of 1.077 g/mL, was acquired from Sigma-Aldrich (St. Louis, MO, USA) to isolate PBMCs from chicken blood. Magnetic beads were used for cell separation with specific antibodies, including anti-FITC, anti-APC, and anti-PE micro-beads. Miltenyi Biotec (Auburn, USA) was the source of all materials, including the MACS assembly, column, and miniMACS starting kits. Mouse anti-chicken antibodies were used for flow cytometry and sourced from Southern Biotech (Birmingham, AL, USA).

### 2.3 Histopathological and immunohistochemical staining

Six control and 12 infected chicks were euthanized using intravenous pentobarbital injection, and samples were collected from the nasal passages, trachea, lungs, kidneys, and proventriculus at 12, 24, and 48 hpi. The beak in front of the nostril and the head were cut off. The nasal sections were fixed in Bouin solution for 72 h (Tamura et al., 1998). The nasal blocks from the histological analysis were sectioned according to specific fractions. The tissue blocks were effectively decalcified using a solution of 4% paraformaldehyde (PFA) and 5% formic acid in PBS. The nasal sections underwent a thorough dehydration process using progressively higher concentrations of ethyl alcohol, were cleared with xylene, and were embedded in high-quality paraffin wax. The paraffin-embedded sections of tissues were precisely prepared by cutting them into 4-µm cross-section slices with a Minux S700 microtome machine. The section was carefully mounted on slides and deparaffinized in xylene. It was rehydrated by transitioning through descending grades of ethyl alcohol and underwent IHC staining to provide detailed insights. The IBV-QX positive antigens were effectively detected through IHC staining, utilizing a particular mouse monoclonal primary antibody sourced from Novus Biological Company. The detection process was further enhanced by employing an HRP-conjugated anti-mouse IgG SABC kit from Beyotime. The slides were subsequently mounted using oleoresin of the balsam. The stained sections were examined using the Olympus BX53 microscope in conjunction with the SC180 digital camera system. The quantification of IBV-QX-positive antigens was statistically measured by using ImageJ.

### 2.4 Immunofluorescence assay

Antigen retrieval was carried out as described, following deparaffinization and rehydration of the paraffin-embedded slides. The slides were kept in a blocking solution (10% PBS, 0.0l g/mL donkey serum (DS), 0.1% BSA, and X-100 Triton). The first antibody was diluted in PBS and 1% DS. The sections were then incubated in the second antibody against IBV-N protein (IB95) (NBP2-31102, NovusBio) for 90 min at room temperature in darkness. Following three washes, conjugated-gold DAPI (P36962, Invitrogen, CA, USA) was dropped and imaged by using a microscope (BS61VS, Olympus).

### 2.5 Flow cytometry

PBMC cell suspensions were carefully collected from each treatment group. The cells were precisely standardized at 1 × 10^6^ cells, ensuring consistency and accuracy for optimal staining results. For both B and T cells, PBMCs were triple-stained using Bu1, CD4, CD45, and MHC II markers. The antibodies used for flow cytometry included FITC-conjugated mouse anti-chicken Bu1, PE-conjugated mouse anti-chicken CD4, APC-conjugated mouse anti-chicken CD45, and PE-conjugated mouse anti-chicken MHCII, incubated for 30 min at 4°C. PBS was used to wash the cells. The positively stained cells were analyzed using FACS (BD FACS Calibur). FlowJo™ v10.9 (BD Life Sciences, USA) was used to analyze the data.

### 2.6 Cell lines and viral infection

NCI-N87 and DF1 cells were successfully sourced from Oricell Bio and ATCC. DF1 cells were cultured in Dulbecco’s Modified Eagle Medium and supplemented with 10% fetal bovine serum (FBS). The NCI-N87 cells were seeded at 4 × 10^5^ cells/cm² in high-quality RPMI-1640 medium (Invitrogen), supplemented with 5% FBS and 1% penicillin–streptomycin (PenStrep; Gibco, Invitrogen).

### 2.7 Transfection of miRNA mimics or inhibitors for the measurement of IBV growth

NCI-N87 cells were seeded in 12-well plates for 24 h, followed by infection with IBV-QX strain for 2 and 24 h and siRNAs’ or their inhibitors’ (200 nM) transfection via Lipofectamine 2000 transfection reagents (Invitrogen). At 24 h post-transfection, cell cultures were harvested to assess gene silencing efficiency.

### 2.8 Real-time quantitative polymerase chain reaction

Total RNA was successfully extracted from both *in vivo* and *in vitro* samples utilizing TRIzol (Invitrogen) and following the manufacturer’s precise protocol (Shah et al., 2025). This robust method ensured high-quality RNA isolation, essential for reliable downstream analyses. The RNA concentration was measured using a Nanodrop spectrophotometer. Complementary DNA (cDNA) was synthesized from RNA using reverse transcription with the HiScript™ qRT SuperMix kit from (Vazyme Biotech Co., Ltd) according to the manufacturer’s manual. Then, qRT-PCR was manipulated by using the SYBR qPCR master Mix kit (Vazyme Biotech Co., Ltd.) on a real-time PCR System ForeQuant F4/F6. All of the amplification primers utilized in this study are listed in **Table 1**. The relative gene expression level was normalized according to glyceraldehyde 3-phosphate dehydrogenase (GAPDH) expression. Each experiment and sample were independently repeated three times and run in technical triplicate. The relative expression was determined using 2^^-ΔΔCt^.

**Table 1:** Quantitative real-time fluorescence PCR primer sequences.

Gene	Primer (5′–3′)	
*chGAPDH*	F	CACACAGAAGACGGTGGATG
	R	TGAGGTGTATTGCTGGCTGT
*hsaGAPDH*	F	GAAGGTGAAGGTCGGAGTC
	R	GAAGATGGTGATGGGATTTC
*IBV-QX*	F	CTGACCTGAGTTGGGGTGAT
	R	GAAGCTGCTGTTGACCTTCC
*IBV-M41*	F	CAAAGGATGCAGCAGTTTCTT
	R	TTCCATATGCAAGCCTCCAGA
*chACE2*	F	ACGCTAGCCGCTTCTCACTAGC
	R	AGCCAATGGATCTGCCAGAA
*chAPN*	F	GACAACGCCTACTCCTCCATTGGC
	R	CACAGAAGGTCTCTCCACCGTGGA
*chAGO2*	F	ACCAGTCTATGCGGAGGTGAA
	R	AACACACTGCGTAGCCATTCC
*chIFITM*	F	TATCCTTTACACCACAGCTGGC
	R	TTGTCCAATCACCTTGTCCTAGC
*chEGFR*	F	TCCTATCCATAAATGCCACAAACA
	R	AAGGCATCCCCTAGAAATGCA
*chDicer*	F	TTTAAACACTGGCTCAGGGAAGA
	R	AAATCCCCCCTGATCTGATAGG
*hsaAGO2*	F	GTTTGACGGCAGGAAGAATCT
	R	AGGACACCCACTTGATGGACA
*hsaDicer*	F	GCTGTGTACGATTGGCTGAA
	R	GGTAGCACTGCCTTCGTTTC
*hsaXPO5*	F	TGGCCACAGAGGTCACCCCC
	R	GGGGCGCAGTGCCTCGTAT
*hsaDrosha*	F	TTGGAACGAGTAGGCTTCGT
	R	CAGGTGCTGTCCTCATCAGA
*hsaIL-1β*	F	CACGATGCACCTGTACGATCA
	R	GTTGCTCCATATCCTGTCCCT
*hsaIL-6*	F	GGTACATCCTCGACGGCATCT
	R	GTGCCTCTTGCTGCTTTCAC
*hsaIL-8*	F	ATGACTTCCAAGCTGGCCGTGGCT
	R	TCTCAGCCCTCTTCAAAAACTTCTC
*hsaIL-4*	F	CTATTAATGGGTCTCACCTACCA
	R	TAAGGCTATAAAAAACTC
*hsaIL-10*	F	CATCAAGGCGCATGTGAACT
	R	GATGTCAAACTCACTCATGGCTTT

### 2.9 Enzyme-linked immunosorbent assay

The concentrations of IL-1β (PI305), IL-6 (PI330), TNF-α (PT518), IL-4 (PI618), and IL-10 (PI528) in NCI-N87 cells were quantified using human enzyme-linked immunosorbent assay (ELISA) kits (Beyotime Biotechnology, China) following the manufacturer’s protocols. All experiments were performed independently three times in triplicate. Data were normalized according to the manufacturer’s protocols.

### 2.10 Western blotting

Western blot (WB) analysis was carried out following the established procedures described in prior studies (Shah et al., 2025). Total protein from the tissues and cell lines was extracted using radioimmunoprecipitation assay (RIPA). A protein assay kit (Boster, China) with bicinchoninic acid (BCA) and protease inhibitors (Merck, USA) was used for quantification. Proteins were separated from the gel by SDS-PAGE and transferred to PVDF paper. After an hour of blocking with skimmed milk, a paper was then incubated with primary antibodies against beta actin (β-actin), (AC026, ABclonal), IBV-N protein (3BN1, Hytest), Argonaute2 (BS70734, Bioworld), Dicer (BS70707, Bioworld), Exportin5 (BS8443, Bioworld), Drosha (BS90429, Bioworld), IL-1β (A16288, ABclonal), IL-6 (EM1701-45, Huabio), TNF-α (11948T, CST), IL4 (ER1706-55, Huabio), and IL-10 (701122, Thermo Fischer Scientific) overnight at 4°C. The enzyme was linked with a secondary antibody at 1-h incubation, and the ECL (enhanced chemiluminescence) kit from Millipore was used to visualize the protein bands. Protein band intensities were quantified and analyzed using ImageJ software (v1.53, National Institutes of Health, USA). Band densities were normalized to β-actin to calculate the relative protein levels. All experiments were performed three times independently.

### 2.11 Quantification and statistical analysis

A statistical analysis approach using Student’s *t*-test and one-way analysis of variance (ANOVA), from the procedure of comparing means, was employed to calculate the significant differences among the groups. Results are expressed as means ± standard deviation (SD), and statistical significance was defined as *p <*0.05. The following symbols denote statistical significance in the figures: ****p < 0.0001, ***p < 0.001, **p < 0.01, *p < 0.05, and ns—nonsignificant. Unless otherwise specified, data from three independent experiments were analyzed.

## 3 Results

### 3.1 Intranasal entry of IBV-QX resulted in tropism for the gastric mucosa and affected the mononuclear immune system

**Figure 1:**
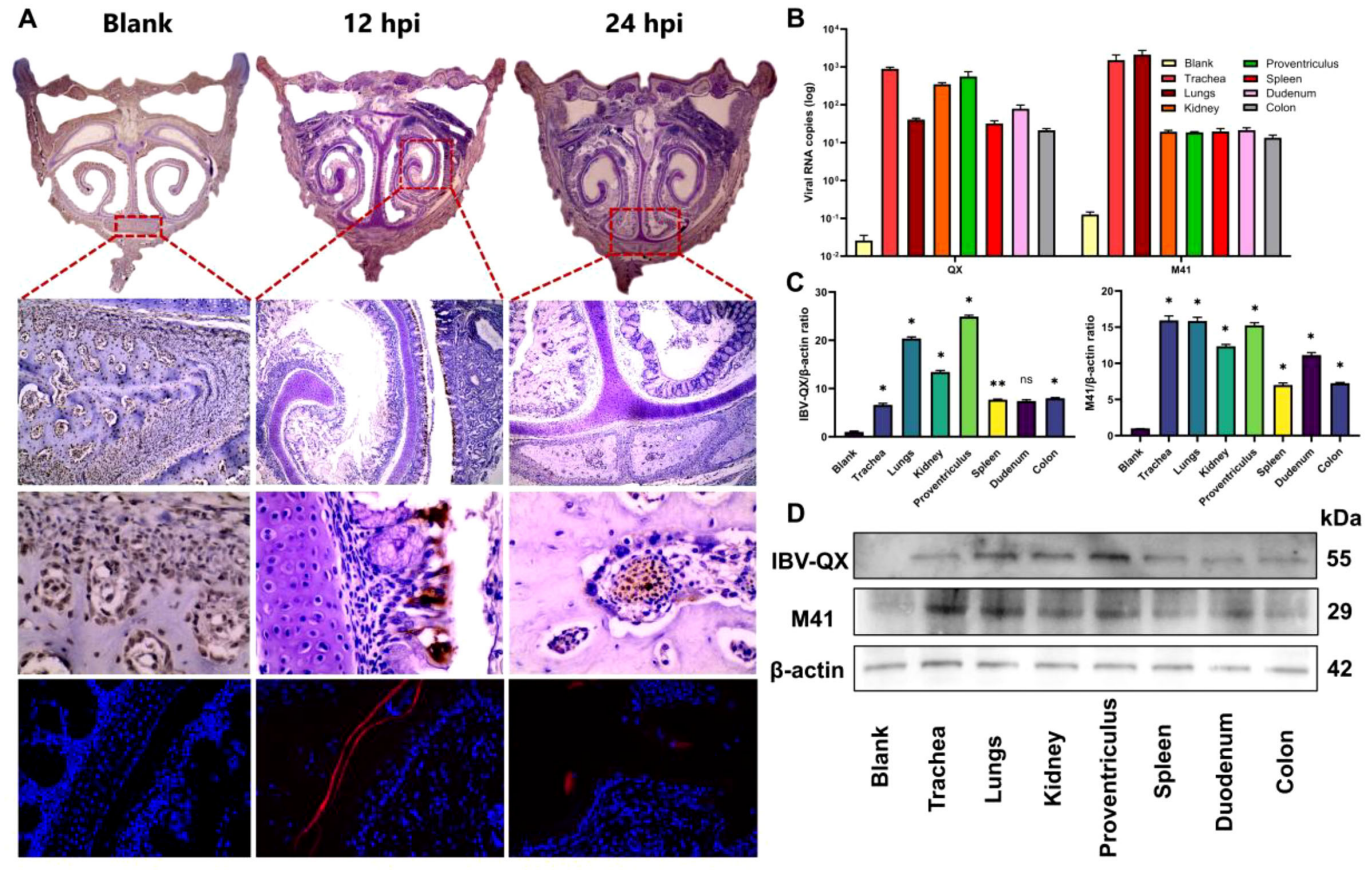
IBV-QX intranasal entry, transmission, and replication in chicken systemic organ tissues and influence on PBMCs. **(A)** Photomicrographs of immunohistochemical and immunofluorescence assay staining of IBV-QX strain in chicken nostrils (areas enclosed in red boxes at 12 hpi—epithelial linings and 24 hpi—nasal-associated lymphoid tissue). **(B)** IBV-QX and M41 mRNA copies in trachea, lungs, kidney, proventriculus, spleen, duodenum, and colon. **(C)** WB quantification of IBV-QX and M41 in trachea, lungs, kidney, proventriculus, spleen, duodenum, and colon. **(D)** WB band intensity of IBV-QX and M41 in trachea, lungs, kidney, proventriculus, spleen, duodenum, and colon. All of the experiments were performed in triplicate (*n* = 3). Significant differences among each group are expressed as non-significant (ns) and *p <0.05 and **p < 0.01. Significance of data presented by one-way ANOVA, with Dunnett’s multiple-comparisons test.

To address the least transmission of IBV strains in the nasal mucosa, inoculation of IBV-QX to chickens intranasally led to the viral antigens in internal organs. At 12 hpi, IBV-QX-positive antigens are found in the epithelial cells of the nasal orifices, and at 24 hpi, these antigens then transmit into the nasal-associated lymphoid tissues (**Figure 1A**). Complete immunofluorescence images are shown in **Supplementary Figure S1**. At 24 hpi, IBV-QX strain RNA replicates highly in the trachea, kidney, and proventriculus (**Figure 1B**), although M41 strain copies are seen highly in the trachea and lungs (**Figure 1B**). IBV-QX proteins exhibit a robust expression in the proventriculus, whereas M41 demonstrates significant presence in the trachea at 24 hpi (**Figures 1C, D**). This marked differential expression underscores the distinct pathogenic mechanisms associated with these viral strains. IBV-QX primarily targets the respiratory tract. Its ability to infect nasal lymphocytes facilitates systemic spread and potentially affects the gastric mucosa through ingestion or viremia, highlighting its broader tropism. This could explain how the virus affects multiple tissues or organs, potentially contributing to broader pathogenesis. Further studies on viral entry mechanisms in these tissues could reveal novel therapeutic targets.

### 3.2 IBV-QX transmission and replication in systemic organ tissues and interference in PBMCs

**Figure 2:**
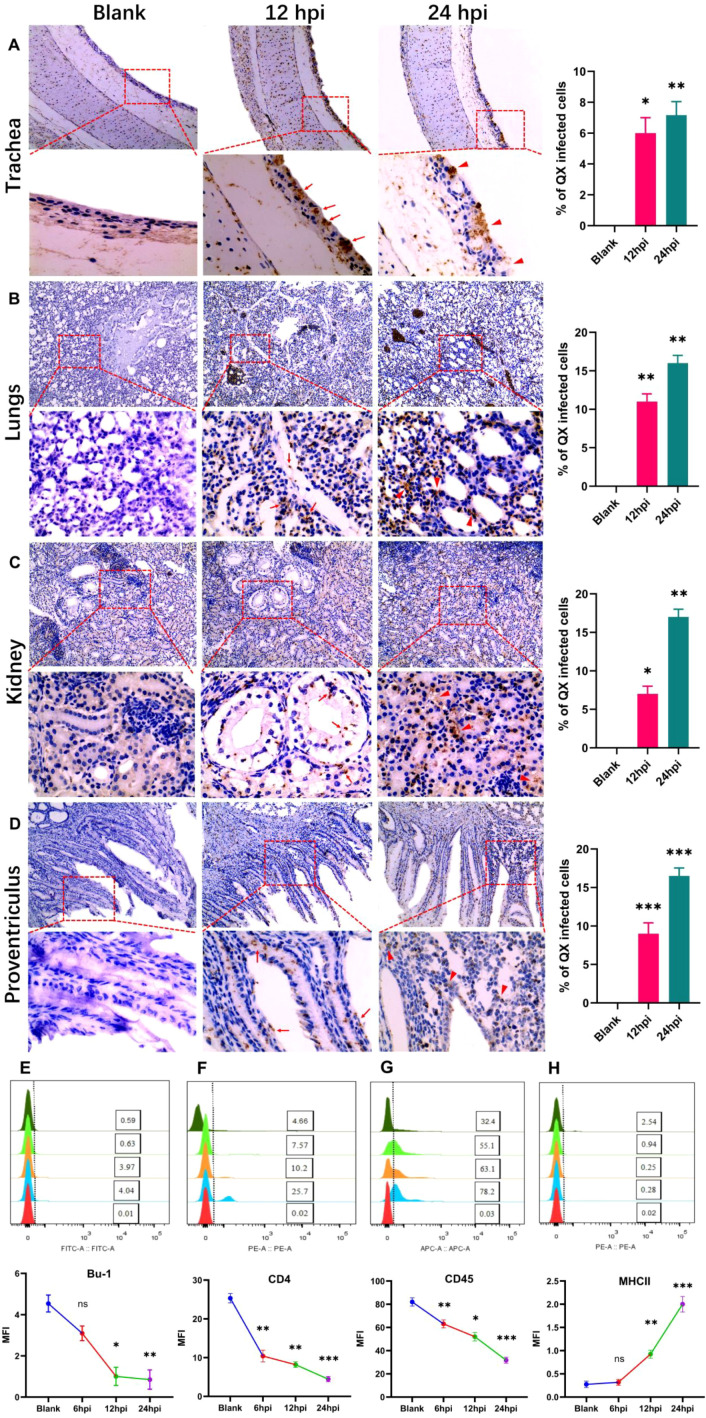
Photomicrographs of IBV-QX transmission and replication in systemic organs by interfering with the defense mechanism. **(A)** Immunohistochemical staining of chicken trachea. **(B)** Lungs, **(C)** kidney, and **(D)** proventriculus at 12 hpi (arrows) and 24 hpi (triangular arrows). B- and T-lymphocytes identified in PBMCs by flow cytometry. **(E)** Bu-1, **(F)** CD4, **(G)** CD45, and **(H)** MHCII. All of the experiments were performed in triplicate (*n* = 3). Significant differences among each group are expressed as non-significant (ns), *p <0.05, **p <0.01, and ***p <0.001. Significance of data presented by one-way ANOVA, with Dunnett’s multiple-comparisons test.

A gammacoronavirus primarily affects chickens, causing infections at least in the respiratory system, renal system, and stomach. At 12 hpi, viral stains appear in trachea pseudostratified ciliated cells and inflammatory cell infiltration (**Figure 2A**). At 24 hpi, desquamation of cells occurs (**Figure 2A**). In the lungs, it accumulates in the capsular mesothelial and connective tissue at 12 hpi. At 24 hpi, it is highly expressed at the alveolar epithelium (**Figure 2B**). Transmission occurs in the kidney at 12 hpi in proximal and distal convoluted tubules (PCT and DCT) at 12 hpi. At 24 hpi, it accumulates in the regions and edges of the renal cortex and renal corpuscles (**Figure 2C**). Previous results demonstrate that QX strain replication occurs in almost all organ tissues, but in the stomach, they are very high because this strain invades mucus-secreting surfaces. At 12 hpi, it is highly expressed in mucus-secreting epithelial cells at the epithelial lining, while at 24 hpi it accumulates highly in mucosal ridges and lamina propria (**Figure 2D**). Here we revealed that IBV-QX affects respiratory lymphocytes and enters the bloodstream, where it transmits and replicates throughout the body by targeting its destination.

Immune B- and T-lymphocytes play a crucial role in eliminating viral infection. We identified B- and T-lymphocytes in PBMC, at least in the infection of chicks with IBV-QX. The reduction in B-lymphocytes (expressing Bu-1) and T-lymphocytes (expressing CD4 and CD45) resulted in viral infections at various time points (**Figures 2E–G**). However, the B-lymphocytes expressing MHC-II levels increase following an intranasal infection with IBV-QX (**Figure 2H**). These results altogether show that alterations in immune lymphocytes occur within 24 h due to high transmission and replication of IBV-QX.

### 3.3 IBV-QX nucleocapsid protein binds and interferes with systemic organs, receptors, and proteins

**Figure 3:**
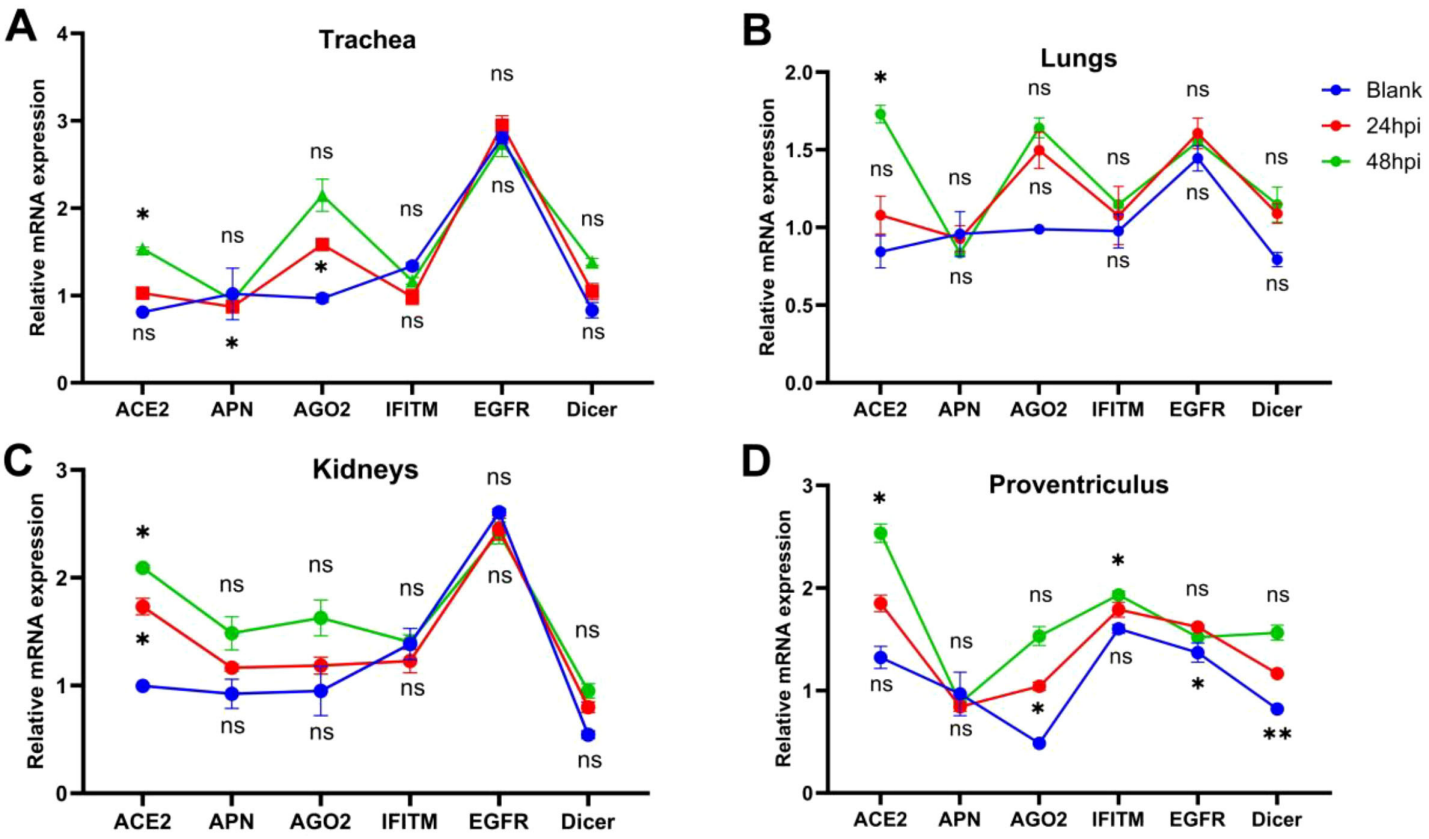
Attachment and interference of IBV-QX with receptors and intracellular proteins. **(A)** Relative mRNA expression of ACE2, APN, AGO2, interferon-induced transmembrane (IFITM), epidermal growth factor receptor (EGFR), and Dicer in trachea. **(B)** Lungs, **(C)** kidney, and **(D)** proventriculus at 12 and 24 hpi. All of the experiments were performed in triplicate (*n* = 3). Significant differences among each group are expressed as non-significant (ns), *p <0.05, and **p <0.01. Significance of data presented by one-way ANOVA, following Bonferroni’s multiple-comparisons test.

IBV-QX binds to sialic-acid-containing α-2,3-linked receptors on the trachea and kidney. The entire deletion of sialylated mucins from tracheal mucosa and kidney tissue completely inhibits the binding of both receptor binding domain (RBD) proteins. This finding underscores the critical role of sialic acids on host tissues in facilitating QX-RBD binding (Bouwman et al., 2020). After binding, the virus enters via endocytosis or membrane fusion, depending on host protease activity. The virus replicates highly intracellularly, causing cell death and inflammation. Here we found that IBV-QX might interact with known receptors in chicken systemic organs such as the trachea, lungs, kidney, and proventriculus. Aminopeptidase (APN) in (kidneys), AGO2 in (trachea, lungs, kidneys, and proventriculus), ACE2 in (trachea, lungs, and kidneys), and Dicer in (lungs and proventriculus) in a time-dependent manner are all considerably upregulated (**Figures 3A–D**). Our findings revealed that IBV-QX nucleocapsid (N) protein interacts with ACE2, AGO2, and Dicer, blocking pre-miRNA nuclear export and processing, thereby suppressing miRNA-mediated antiviral responses. IBV-QX may disrupt this pathway, impairing pre-miRNA transport.

### 3.4 IBV-QX suppresses the expression of key components of the miRNA biogenesis pathway

**Figure 4:**
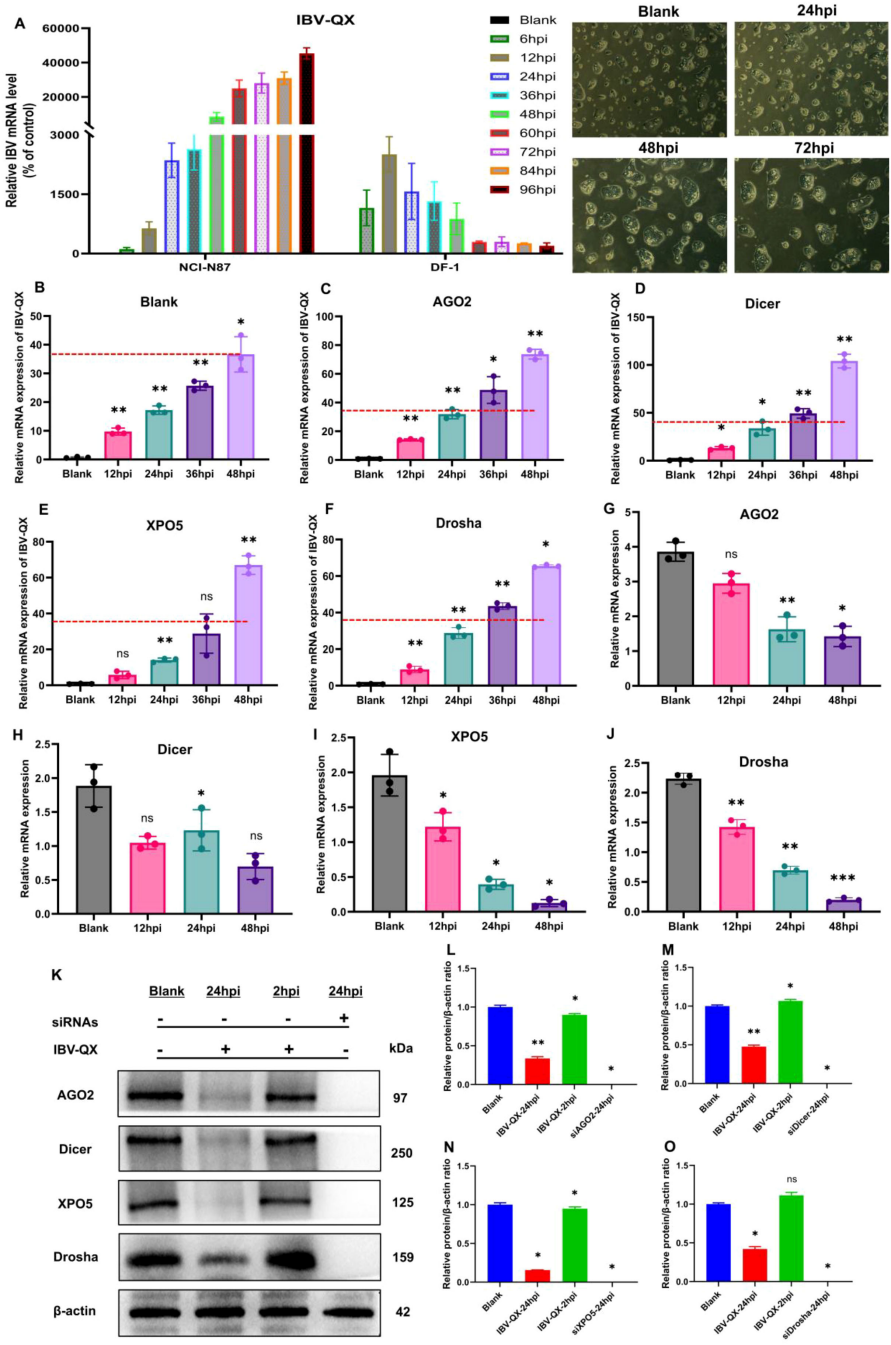
IBV-QX interferes and hinders the synthesis of the miRNA biogenesis pathway. **(A)** Photomicrographs of mRNA levels of IBV-QX in NCI-N87 and DF-1 cells. Relative mRNA levels of IBV-QX in NCI-N87 cells transfected for 48 h with inhibitors. **(B)** Blank, **(C)** AGO2, **(D)** Dicer, **(E)** XPO5, and **(F)** Drosha. Relative mRNA levels of biogenesis factors, **(G)** AGO2, **(H)** Dicer, **(I)** XPO5, and **(J)** Drosha in NCI-N87 cells infected with IBV-QX. **(K)** WB protein band intensities of AGO2, Dicer, XPO5, and Drosha transfected with IBV-QX and miRNA inhibitors for 24. WB relative protein quantification of biogenesis factors, **(L)** AGO2, **(M)** Dicer, **(N)** XPO5, and **(O)** Drosha. All of the experiments were performed in triplicate (*n* = 3). Significant differences among each group are expressed as non-significant (ns), *p < 0.05, **p <0.01, and ***p < 0.001). Significance of data presented by one-way ANOVA, with Dunnett’s multiple-comparisons test.

An *in vitro* study demonstrates that IBV-QX replicates highly in the gastric mucosa. To validate our *in vivo* findings, we chose the gastric epithelial cell line along with the DF-1 cell line and transfected it with the IBV-QX strain. IBV-QX replication in DF-1 cells decreases after 12 hpi, whereas it peaks at 12 hpi (**Figure 4A**). However, the QX strain replicates highly in NCI-N87 cells in a time-dependent manner. This study aims to understand the cellular pathways implicated in IBV-QX replication, specifically targeting the miRNA biogenesis pathway to investigate its role in regulating the host immune response during viral replication. We target the miRNA biogenesis pathway to check whether it regulates the host immune response in viral replication. In NCI-N87 gastric cells, we first employ inhibitors to silence (si-miRNAs) host transcription factors like Drosha, Dicer, AGO2, and XPO5 for 24 h. Then, we transfected the cells with QX strain for 24 hpi. By using miRNA inhibitors, the mRNA replication of QX strain increases almost once in Drosha, AGO2, and XPO5 and twice in Dicer compared to the blank (**Figures 4B–F**). Here only the cells transfected with QX decrease the mRNA levels of miRNA biogenesis transcription factors in a time-dependent manner, except Dicer (**Figures 4G–J**). QX transfection inhibits the protein synthesis of these biogenesis factors (Drosha, Dicer, AGO2, and XPO5) in gastric cells at 24 hpi (**Figures 4K–O**). To validate these results, we also checked the protein expressions at 2 hpi. We found the protein levels of Dicer (**Figure 4M**) and Drosha (**Figure 4O**) to increase, whereas AGO2 (**Figure 4L**) and XPO5 (**Figure 4N**) decrease slightly. Here we use inhibitors by silencing miRNA biogenesis factors in cells to compare our findings. Specific studies on IBV-QX are limited; it likely disrupts miRNA biogenesis similarly to other coronaviruses by interfering with the processing, exporting, or functioning of miRNAs to promote viral survival. Further research is needed to pinpoint the exact mechanisms. These findings altogether highlight that QX inhibits the synthesis of key components of the miRNA biogenesis pathway and ultimately replicates highly in gastric cells. Our findings suggest that the IBV-QX strain may be potentially involved in cross-species transmission.

### 3.5 Evaluating the pro- and anti-inflammatory cytokine responses

**Figure 5:**
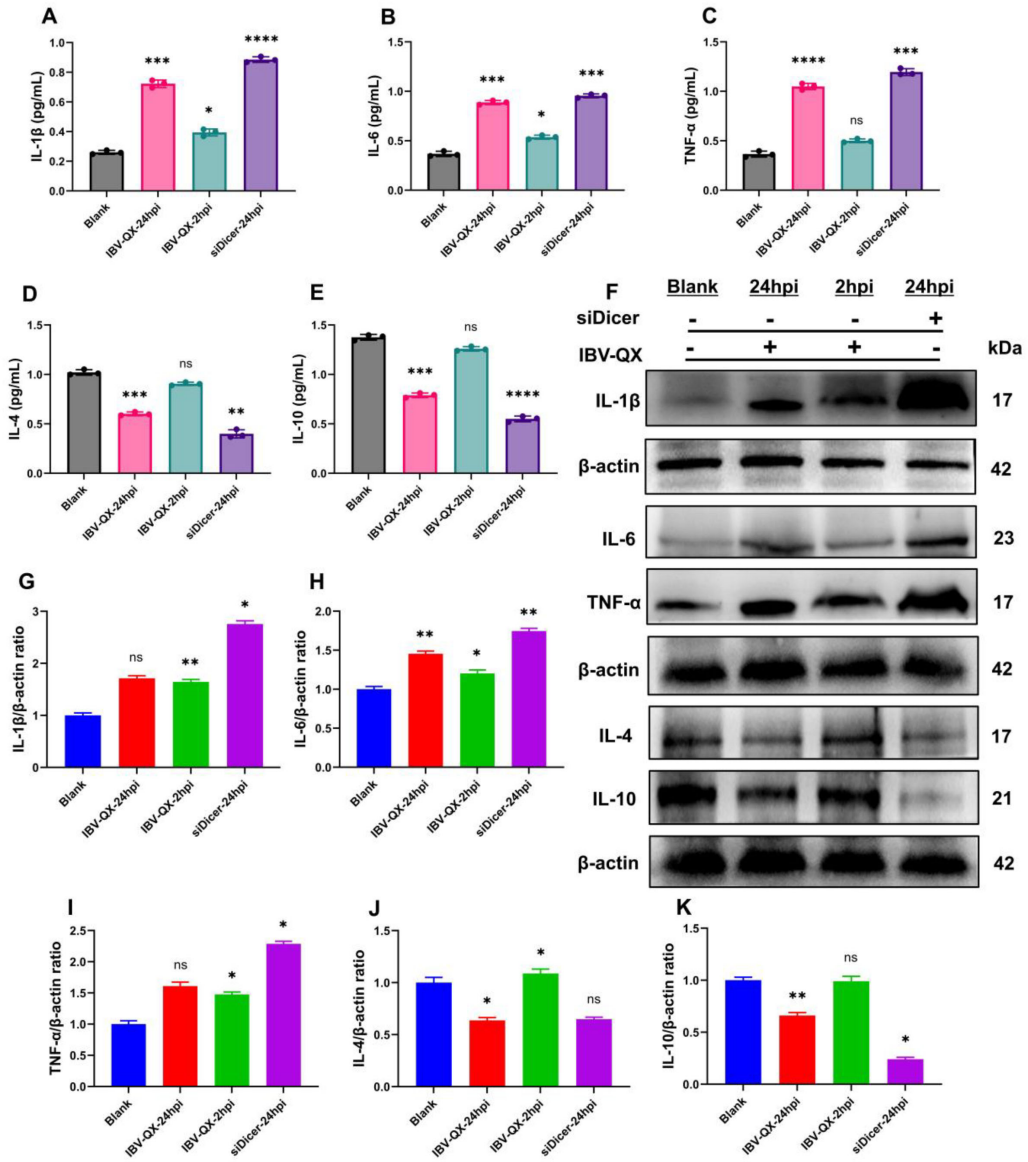
IBV-QX and knockdown of the miRNA biogenesis pathway activate inflammatory cytokines and suppress anti-inflammatory molecules. Relative inflammatory and anti-inflammatory expression levels of **(A)** IL-1β, **(B)** IL-6, **(C)** TNF-α, **(D)** IL-4, and **(E)** IL-10 in gastric NCI-N87 cells. **(F)** Representative WB band intensities analysis of IL-1β, IL-6, TNF-α, IL-4, and IL-10 in NCI-N87 cells. Relative protein quantification of **(G)** IL-1β, **(H)** IL-6, **(I)** TNF-α, **(J)** IL-4, and **(K)** IL-10. All of the experiments were performed in triplicate (*n* = 3). Significant differences among each group are expressed as non-significant (ns), *p <0.05, **p <0.01, ***p <0.001, and ****p < 0.0001. Significance of data presented by one-way ANOVA, with Dunnett’s multiple-comparisons test.

We evaluated the inflammatory and anti-inflammatory cytokines in NCI-N87 cells infected with IBV-QX at 2 and 24 hpi. Likewise, we also use a Dicer inhibitor to silence their synthesis in gastric cells. Moreover, our ELISA analysis confirmed that the levels of three key pro-inflammatory cytokines (IL-1β, IL6, and TNF-α) are activated (**Figures 5A–C**), and the anti-inflammatory cytokines (IL-4 and IL-10) are suppressed (**Figures 5D, E**) with QX transfection at 2 and 24 hpi. Similarly, these pro-inflammatory cytokines increase (**Figures 5A–C**), and the anti-inflammatory cytokines decrease (**Figures 5D, E**) when the miRNA biogenesis key transcription component Dicer is silenced in gastric cells at 24 hpi. Similarly, the mRNA levels of IL-1β, IL6, and TNF-α increase in gastric cells transfected with QX strain at 24 hpi and silenced with Dicer as shown in **Supplementary Figures S2A–C**. Furthermore, we validate these cytokine protein expressions with WB, which are parallel to the ELISA and mRNA findings. At 24 hpi, QX transfection and Dicer inhibition activate the levels of inflammatory cytokines in gastric cells (**Figures 5F–I**), whereas the levels of anti-inflammatory molecules’ protein decrease with QX transfection and the inhibition of the Dicer synthesis in NCI-N87 cells at 24 h (**Figures 5F, J, K**). Consequently, these results conclude that the IBV-QX strain highly replicates in gastric cells by inhibiting the miRNA biogenesis pathway, leading to the activation of inflammatory and suppression of anti-inflammatory cytokines, respectively.

## 4 Discussion

We examined the rate of IBV-QX replication primarily in chickens, which results in nasal-associated lymphoid tissue at 24 hpi. IBV-QX demonstrates extensive tropism, highly infecting the gastric mucosa, renal tissue, and respiratory epithelium, which encourages systemic dissemination. Here the QX strain exhibits a piece of evidence suggesting its ability to infect immune cells and other tissues, including the nasal-associated lymphoid tissue (NALT) and the gastric mucosa. Other IBV strains enter host cells via the spike protein, which binds to host receptors like sialic acid and ACE2 and mediates membrane fusion. Further research is needed to clarify the exact receptors and immune evasion mechanisms involved. The capacity of a virus to disseminate beyond its initial site of entry depends on several key factors, including its mode of transport, tropism for specific tissues, and the host immune defenses. Viremia has been suggested as a potential dissemination route for IBV (Hofstad and Yoder, 1966). Recent research has increasingly highlighted the critical role of mononuclear cells, particularly PBMCs, in facilitating viral dissemination within the body (Amarasinghe et al., 2017; Reddy et al., 2016). Our flow cytometry results showed that QX strain inhibits the synthesis of B- and T-lymphocytes at the utmost earliest time of duration post-infection.

IBV-QX demonstrates exceptional replication efficiency in the proventricular tissue (Yudong et al., 1998; Laconi et al., 2020; De Wit et al., 2011). Viral loads in the proventriculus can reach 10^8^ to 10^9^ copies/μg RNA in acute infections (Promkuntod et al., 2014). Preferential binding to α-2,3-linked sialic acid receptors abundant in proventricular epithelium causes lymphocytic infiltration and glandular atrophy (Cook et al., 2012; Bartel, 2018). Our findings show that some receptors are highly expressed in systemic organs of QX-infected chickens, highlighting their involvement in the replication and pathogenicity of QX-like strains.

Gene expression is regulated by miRNAs in humans, which influence a broad range of physiological and pathological processes. Their roles extend to critical areas, including developmental biology, cancer progression, viral infections, and the antiviral immune response mechanisms. Understanding the impact of miRNAs is essential to uncover the complexities of these processes and advance medical science (Mousavi et al., 2021). The suppression of factors involved in the miRNA biogenesis pathway is thought to be the primary cause during COVID-19 infection. This result demonstrates a critical connection that implies coronaviruses may interfere with the miRNA biogenesis pathway (Schütz and Sarnow, 2006; Mockenhaupt et al., 2011) and validates the findings that AGO2, Dicer, and Drosha were considerably decreased in COVID-19 victims compared to the control participants (Casseb et al., 2016). A549 cells infected with dengue virus serotype 4 lead to a decrease in Dicer, Drosha, and DGCR8 mRNA, suggesting the modulation of host miRNA processing machinery (Matskevich and Moelling, 2007). Infection of Vero and A549 cells with influenza virus-A leads to a reduction in both the proteins and mRNA of Dicer (Kameka et al., 2014). However, numerous viruses engage with Dicer and Drosha to generate viral miRNAs or modulate viral transcripts (Cullen, 2013; Kincaid et al., 2012; Pfeffer et al., 2004). Nonstructural protein 15 (NSP15) of IBV possesses an endonuclease function, a trait conserved across coronaviruses (Zhao et al., 2021). IBV-NSP15 plays a key role in host shutoff in both mammalian and avian cells (Zhao et al., 2024). Beyond evading the host’s miRNA-regulated defenses, viruses also hijack these pathways to facilitate their replication and drive pathogenesis.

**Figure 6:**
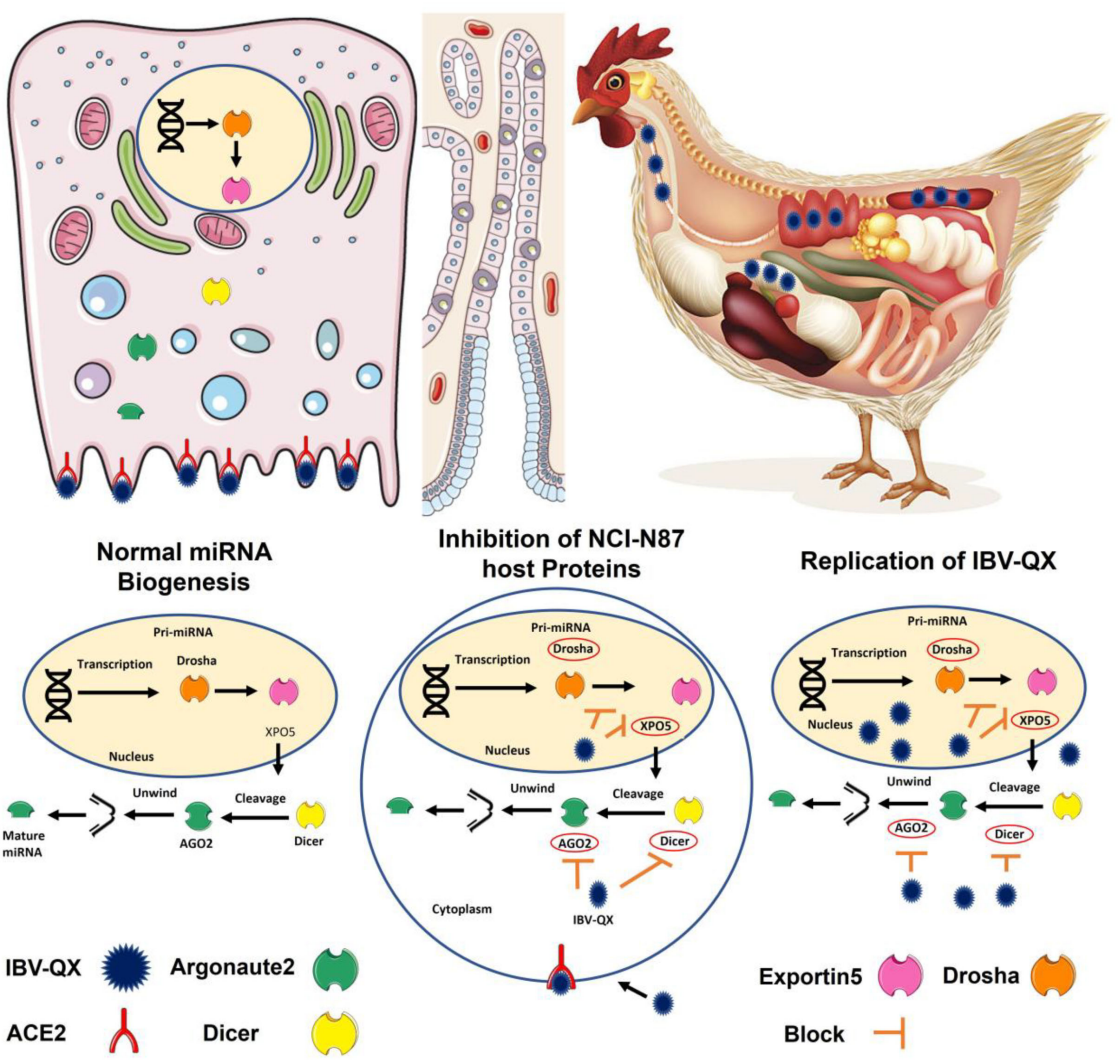
This hypothetical model provides key insights into how IBV-QX hijacks host miRNA machinery to promote infection and inflammation. The QX strain circulates in the lymphatic system, suggesting systemic dissemination beyond the primary infection site. Similarly, QX-RBD may optimize binding to specific receptors, which aligns with known mechanisms of some coronaviruses. Viral entry into host cells is mediated by specific receptors, enabling the virus to hijack cellular machinery. The QX strain interferes with the translation of key miRNA biogenesis proteins (AGO2, Dicer, XPO5, and Drosha). By inhibiting miRNA biogenesis, the virus may evade host-RNAi-based antiviral defenses. The knockdown of RNAi-related genes (e.g., AGO2, Dicer) could enhance viral replication and upregulate pro-inflammatory pathways, contributing to tissue damage. This is a fascinating and potentially significant finding regarding the interaction between the IBV-QX strain and the host miRNA biogenesis pathway in gastric cells.

The IBV-QX effect on the miRNA biogenesis pathway is not extensively documented in the literature. Based on the general knowledge of how viruses, particularly coronaviruses like IBV, interact with the host miRNA biogenesis pathway, we can effectively uncover potential mechanisms that may significantly impact viral and host interactions. It is possible that QX-like strain generates small ssRNAs mimicking cellular miRNAs, which may alter the activity of host key RNA-processing machinery such as Drosha–DGCR8 and Dicer–TRBP complexes. This mimicry could serve a dual purpose, suppressing antiviral miRNAs by competing for processing machinery and enhancing viral replication by co-opting the miRNA biogenesis pathway to prioritize viral small RNA synthesis (**Figure 6**). This leads to altered miRNA maturation and disrupts their processing, helping the virus evade immune detection. IBV increases inflammatory cytokines (IL-1β) in both the trachea and lungs (Guo et al., 2008). The observation that IL-1β peaks at 72 hpi with mass-type IBV immunization aligns with findings from several studies (Wang et al., 2006). The rapid upregulation of IL-1β gene expression in response to viral infections is a well-documented phenomenon in mammalian studies, often observed as early as 6 hpi (Lawrence et al., 2013; Poeck et al., 2010). IBV promoted the expression of nearly all related proteins in macrophages, with only a slight suppression observed at earlier stages. This regulation allows IBV to exert its biological functions across various host responses. Additionally, the dynamic changes in gene expression were closely linked to virus replication (Sun et al., 2021). The enhanced binding of QX-RBD to host cell receptors, which may facilitate viral entry and accelerate replication, altogether presents challenges for vaccine development. The lack of detailed knowledge about these mechanisms complicates efforts to mitigate zoonotic risks and design effective vaccines or therapeutics. Addressing these gaps through further research is essential to improve the strategies to control IBV outbreaks in poultry. While this study provides valuable insights into IBV-QX ability to evade early immune defenses and establish persistent infections, the precise mechanisms by which the virus suppresses miRNA biogenesis factors are not fully elucidated. Additionally, the specific pathways through which RNAi-related gene knockdown enhances viral replication and triggers inflammatory responses remain unclear. A deeper understanding of these interactions is needed to clarify their role in viral pathogenesis and immune evasion strategies.

## 5 Conclusions

Our findings demonstrate that IBV-QX can effectively transmit and replicate in chicken mucosal organs by evading initial mucosal immunity, which leads to prolonged infection or secondary complications. It inhibits the synthesis and translation of miRNA biogenesis factors. It stimulates inflammation by replicating robustly when RNAi-related gene knockdown occurs, suggesting a viral evasion mechanism that exacerbates infection and inflammatory responses.

## Data Availability

The original contributions presented in the study are included in the article/**Supplementary Material**. Further inquiries can be directed to the corresponding authors.
