# Supplementary material for: Infectious bronchitis-virus-like QX strain transmission, pathogenesis, replication, and host miRNA biogenesis pathway hijacking mechanism
Source: Front Cell Infect Microbiol. 2025 Sep 1;15:1645086. doi: 10.3389/fcimb.2025.1645086 (PMC12434004; doi:10.3389/fcimb.2025.1645086)
Supplement: Supplementary file 1 [file DataSheet1.docx]

***Supplementary data***


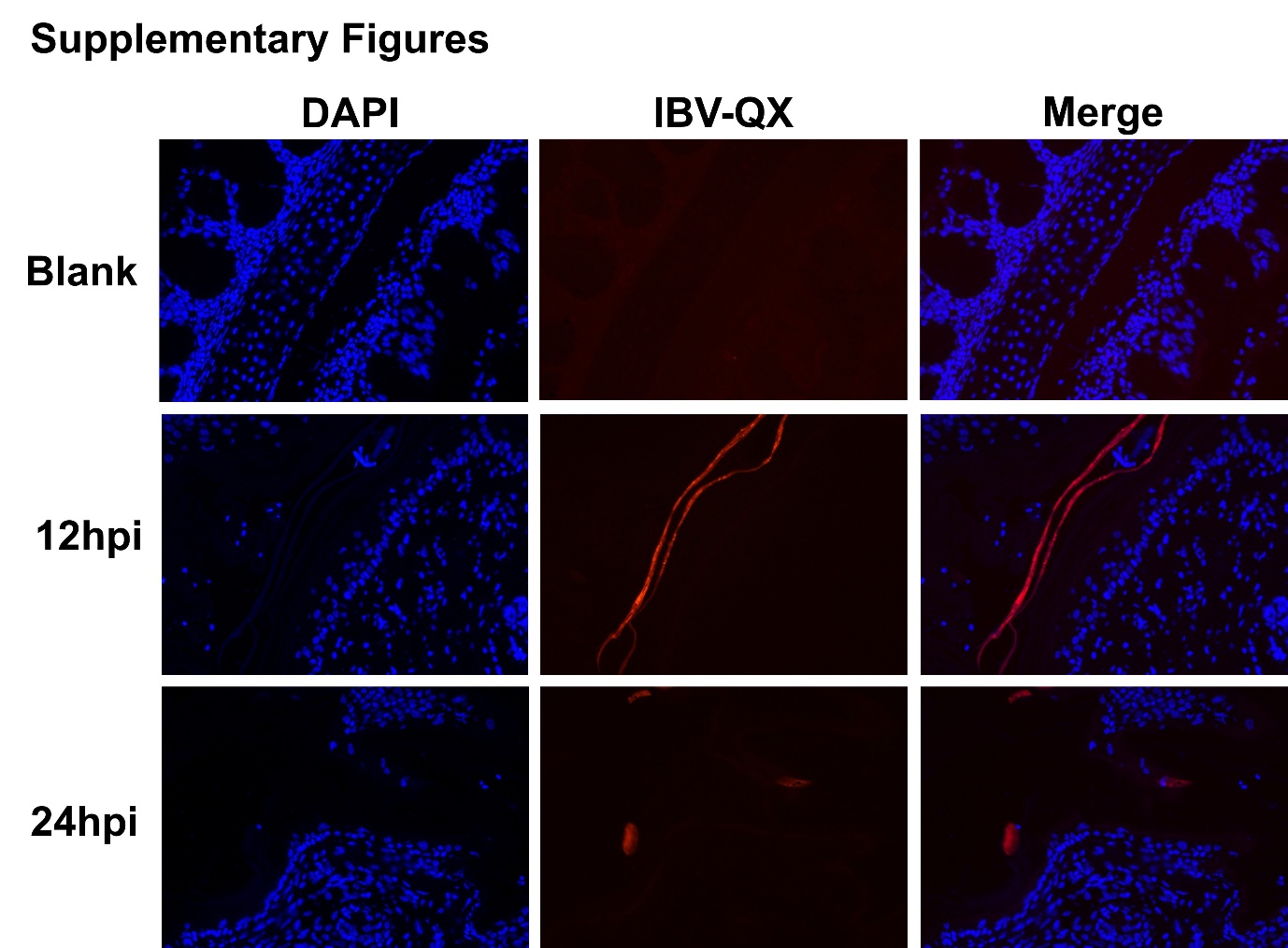


**Supplementary Figure S1.** Representative immunofluorescence staining images of nasal orifices of blank, 12 and 24 hpi of IBV-QX strain. At 12 hpi, QX strain (red) appearance on nasal epithelial linings. At 24 hpi, QX strain transmission occurs on nasal associated lymphoid tissue.


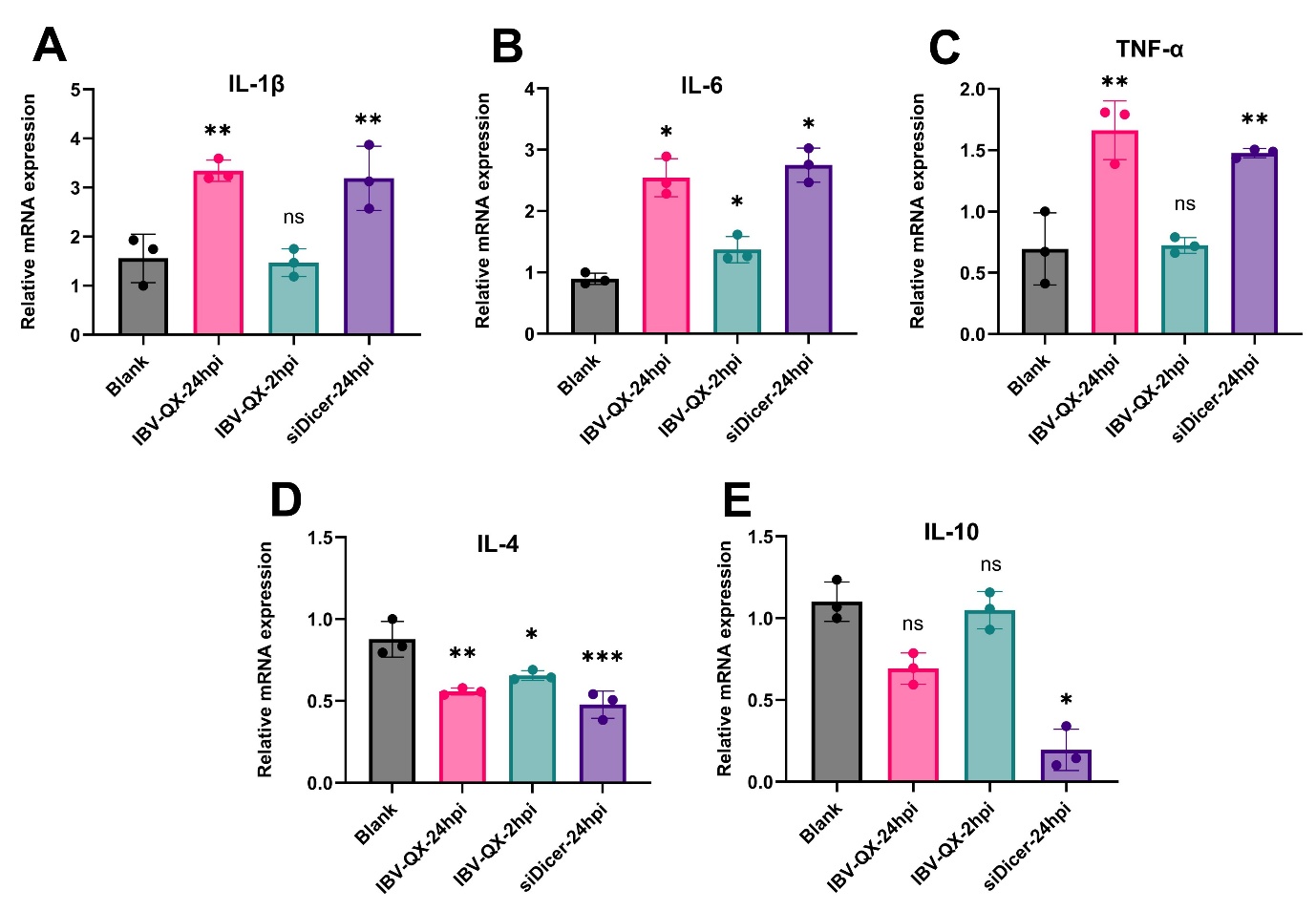


**Supplementary Figure S2.** Representative mRNA levels of inflammatory, **A)** IL-1β, **B)** IL-6, **C)** TNF-α and anti-inflammatory cytokines, **D)** IL-4 and **E)** IL-10 in NCI-N87 cells transfected with IBV-QX and inhibited the synthesis of dicer.
